# Screening for Y-chromosome microdeletions in a population of infertile males in the Gaza Strip

**Published:** 2009-10-20

**Authors:** Ashraf J. Shaqalaih, Masood S. Abu Halima, Mohammed J. Ashour, Fadel A. Sharif

**Affiliations:** Medical Technology Department, Islamic University of Gaza, Gaza-Palestine

**Keywords:** Idiopathic male infertility, A/oligozoospermia, Y-chromosome microdeletions, *AZFc* partial deletion

## Abstract

Infertility is an extraordinary public health problem in the Arab world, as it affects about 15% of couples seeking children. The male partner is responsible for infertility in approximately half of these cases. Classic microdeletions of the Y-chromosome involving the azoospermia factor (*AZF*) regions are known to be associated with spermatogenic impairment, and non-obstructive azoospermia must be differentiated on the basis of endocrine evaluation and testicular biopsy. Partial AZFc deletions remain controversial because there is no clear agreement regarding their role in spermatogenic failure. In the current study, 50 fertile males (controls) and 125 patients with primary idiopathic male infertility were studied in order to describe the frequency of Y-chromosome mirodeletions among male infertility patients in the Gaza Strip-Palestine area. No Y chromosome classical microdeletions could be detected in any of the 125 infertile men, suggesting that ethnic factors, genetic background, and Y chromosome haplogroups are key factors in such deletions. On the other hand, six gr/gr and one b1/b3 *AZF*c partial deletions were detected in the infertile population. The gr/gr deletion was also noted in relatives of four of the six patients with this deletion, and in one of the fertile controls. In conclusion, our study shows that the incidence of Y-chromosome microdeletions in our population is rare; these data suggest that other genetic, epigenetic, nutritional and/or local factors are responsible for impairments in semen parameters observed in this Gazan population. We further hypothesise that the gr/gr deletion is not associated with male infertility, at least in this sub-group.

## Introduction

Male causes of infertility are found in about 50% of couples struggling with infertility [1,2]. Reduced male infertility can be a result of congenital and/or acquired abnormalities. Frequently, however, male infertility is difficult to diagnose and about 60–75% of cases remain idiopathic. These idiopathic cases present with no previous family history associated with fertility problems and have normal findings on physical examination [3]. Interstitial and terminal deletions in *AZF*a, or *AZF*b, or *AZF*c alone or in any combination of the Y-chromosome long arm (Yq) are all associated with dramatic nonobstructive spermatogenic failure. Therefore, there is a clear connection between deletion/s at particular *AZF* loci and male infertility [4–12]. These gross “microdeletions” are associated with divergent testicular histological profiles, ranging from Sertoli cell-only syndrome (SCOS), hypospermatogenesis (HS) to spermatogenic arrest (SGA) [13].

Apart from infertility, men presenting with *AZF* microdeletions appear otherwise healthy. These microdeletions are usually detected by performing sequence tagged site (STS) based PCR techniques on patient peripheral blood genomic DNA. In addition to standard GTG karyotyping, Y-chromosome microdeletion (by PCR) is a mandatory test in the evaluation of the azoo/oligozoospermic patient. Worldwide, the Y-chromosome microdeletion assay has become a routine test, and current research indicates that about 10% cases of idiopathic azoo/oligozoospermia may be due to deletion in *AZF.* Disruption of AZF therefore can be viewed as the most common molecularly diagnosable cause of spermatogenic failure in the setting of nonobstructive azoospermia or severe oligozoospermia [7,12,14–19]. Previous investigators have reported that complete deletion of the entire *AZF*c region (spanning 3.5 Mb of the Y-chromosome) is the most common known genetic cause of human male infertility [11, 15–18, 20–23].

*AZFc* partial deletions probably occur through non-allelic homologous recombination events between amplicons within the *AZFc* region [24–26]. These recombinant events can yield different *AZFc* deletion patterns (*e.g*., gr/gr, b1/b3 and b2/b3) and are characterized by particular region-specific STSs [17, 20, 25, 27–29]. While there is no consensus on whether partial *AZFc* deletions affect spermatogenesis, some authors have suggested that such deletions represent a risk factor for male infertility [28–30]. Other authors, however, found no association between certain *AZFc* partial deletions and infertility [31–33].

The association between Y-chromosome microdeletions and male infertility has not been specifically studied in patients from the Gaza Strip (Palestine) until now. To do so we employed a PCR STS-based technique to detect Y-chromosome microdeletions in a group of azoo/oligozoospermic Gazan infertile patients.

## Materials and Methods

### Study Population

A total of 125 infertile Palestinian males residing in Gaza Strip with non-obstructive sperm impairments were evaluated. These patients were confirmed to have non-obstructive azoospermia or oligozoospermia by endocrine evaluation and testicular biopsy. These patients had cryptozoospermia (sperm count <0.1M/ml), severe oligozoospermia (sperm count>0.1 and <5M/ml), or oligozoospermia (sperm count 5–10M/ml) and were recruited from assisted reproduction centers and private infertility clinics between June 2006 and August 2008. Patients presented with primary infertility and having sperm counts less than 10 M/ml on at least 2 consecutive occasions were included for study. The control group consisted of 50 Gazan men with proven fertility, defined as conceiving at least one child without medical assistance. All study subjects provided written informed consent in compliance with the Helsinki Ethical Committee in Gaza.

### DNA Extraction and PCR

Approximately 2 ml venous peripheral blood samples were collected in K3-EDTA tubes. Genomic DNA from patient and control samples was extracted and purified by using Wizard^®^ Genomic DNA Purification Kit (Promega) following the manufacturer protocol. Microdeletion analysis of the Y-chromosome Yq region involved two components. The first step aimed to detect *AZF*a, *AZF*b and *AZF*c complete microdeletions. 13 STSs (*AZF* loci) mapped at intervals 5 and 6 on the long arm of the Y chromosome were used: sY746, sY84, sY86, and DBY1 for *AZF*a, sY117, sY125, sY127, sY131, and sY134 for *AZF*b, and sY152, sY272, sY254 and sY255 for *AZF*c. In addition, SRY (sex determining region on Y) gene and X/Y homologous gene pair zinc-finger X (*ZFX*) and Zinc-Finger Y (*ZFY*) primers were used as positive internal controls to detect amplification failures in case a microdeletion was detected. In the second step, when step one does not show any *AZF* deletion for any patient, we looked for *AZFc* partial deletions using sY1291, and sY1191 primer sets. Sequences of all primer pairs and expected size of their products are shown in Table 1.

PCR was carried out in a monoplex fashion for each primer set. PCR was carried out in a 0.2ml PCR Microfuge tube in a 20μl reaction volume containing: 2μl template genomic DNA (100–200ng), 10μl PCR Master mix (Promega, Madison, USA) 1.5 μl (2 μmol) each primer and nuclease free sterile water to 20 μl. The amplification reaction was performed in a programmable thermal cycler. Amplification was started with initial denaturation step at 94°C × 15min, followed by 35 sequential cycles each including 60sec denaturation at 94°C, 60sec primer annealing at 57°C and 60sec extension at 72°C. The protocol was followed by a final extension step at 72°C × 10min followed by cooling to 4°C until electrophoretic detection.

For detecting *AZFc* partial deletions, the same reaction mixture and volume were used as above, but instead employed different primer sets. The following PCR protocol was employed: 5min initial denaturation (94°C), followed by 35 sequential cycles of 30sec denaturation (94°C), 45sec primer annealing (61°C) and 45sec extension (72°C). This was followed by an extension step of 7min at 72°C with subsequent cooling to 4°C until electrophoretic detection. In case of detecting a partial deletion, available first degree relatives were also tested for the presence of that deletion. Note: Testing for origin of *AZFc* partial deletions among family members of study patients (*i.e*., inherited vs. *de novo*) was possible in only four cases.

Positive and negative controls were run concurrently with each patient sample. Female and fertile male DNA samples were used as negative and positive controls, respectively. Water instead of genomic DNA was used as blank to check for any DNA contamination.

The PCR product was added to the loading dye, mixed and run on a 2 % (w/v) agarose gel containing 0.5 μg/ml ethidium bromide in 1xTris Acetate EDTA (TAE). In addition, a 100bp DNA ladder was always run concurrently with each electrophoretic run to confirm product size. After electrophoresis at 70 volts × 45min, results were visualized and recorded using a documentation system (Vision, Scie-Plas Ltd, UK).

### Statistical Analysis

Partial deletion frequencies in the patient and control groups were compared using the Chi square; *p*<0.05 was considered statistically significant.

## Results

### Full AZF Microdeletions

**Figure 1.**
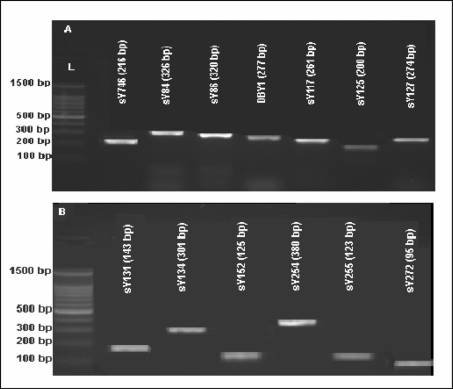
Representative ethidium bromide-stained agarose gels for detection of classic (full) *AZF* microdeletions (case #83). The STSs and the PCR product sizes are indicated above each band. L: 100 bp DNA Ladder.

No complete (classic) Y-chromosome microdeletions in *AZFa*, *AZFb* or *AZFc* were detected among the 125 infertile men included in this study. An example of PCR products confirming the lack of classical Y-chromosome microdeletions is shown in Figure 1.

### AZFc Partial Deletions in Patients and Controls

In order to detect partial *AZFc* deletions our goal was to detect the unique fragments flanking the *DAZ1/DAZ2* doublet at the u3 segment (proximal) and the P2/P1 palindrome junction (distal), corresponding, respectively, to sY1191 and sY1291 as described previously [17,18,39]. The patterns shown in Table 2 were used for assigning the different partial *AZFc* deletions.

**Figure 2.**
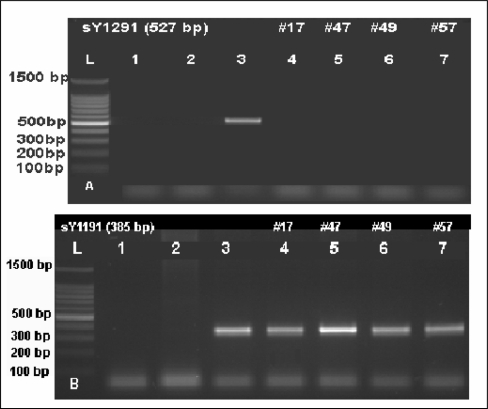
Ethidium bromide-stained agarose gel demonstrating selected gr/gr deletions. Upper panel “A”: sY1291 STS PCR products show absence of sY1291 (527 bp) in gr/gr deletion cases #17, 47, 49, and 57 (lanes 4–7). Lower panel “B”: sY1191 STS PCR results showing presence of sY1191 (385 bp) in the same gr/gr deleted cases (lanes 4–7). L= 100 bp DNA ladder, Lane 1: negative control (water), Lane 2: negative control (female DNA), Lane 3: positive control (fertile male DNA).

**Figure 3.**
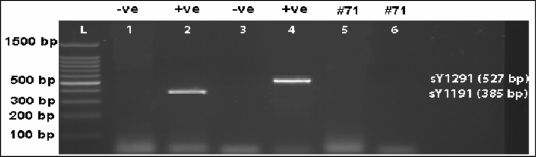
Ethidium bromide-stained agarose gel photo for case# 71, where b1/b3 partial *AZFc* deletion was identified. L: 100 bp DNA Ladder, Lane1: sY1191 negative control (female DNA), Lane2: sY1191 positive control (fertile male DNA), Lane3: sY1291 negative control (normal female DNA), Lane 4: sY1291 positive control (fertile male DNA), Lane 5: sY1191 for case# 71, Lane 6: sY1291 for case# 71. Note that both sY1191 and sY1291 are absent in case# 71, consistent with b1/b3 partial *AZFc* deletion.

In total, seven (5.6%) of 125 infertile men investigated had partial deletions within the *AZFc* region. In particular, we found two different patterns of partial *AZFc* deletions; the gr/gr (6/125, 4.8%) and the b1/b3 (1/125, 0.8%) deletions. One man with gr/gr deletion was oligozoospermic (sperm count 6.4 M/ml), one was severely oligozoospermic (sperm count 0.7 M/ml), but the others demonstrated total azoospermia. The patient with b1/b3 deletion was severely oligozoospermic (sperm count 2.6 M/ml). Testicular biopsy reports were available only for three of the seven patients with partial deletions of the *AZF*c region. One patient had Sertoli cell-only syndrome (SCOS), one had spermatogenic arrest and one patient had severe hypospermatogenesis. One subject (1/50, 2.0%) in the control group proved to have gr/gr deletion, which was the only pattern of partial deletions of the *AZF*c region observed in the control group. The frequency of partial deletions in experimental and control groups is given in Table 3. The sY1291 and sY1191 STS PCR results for selected patients with partial *AZFc* deletions are shown in Figures 2 and 3.

The gr/gr deletion was present in both infertile (6/125, 4.8%) and control (1/50, 2%) groups. No statistically significant difference in the frequency of this deletion was found between the two groups (*p*=0.409). The deletion frequency of b1/b3 in the infertile group was 0.8% (1/125), while it was not observed at all in the control group. The difference in deletion frequency between the two populations also was not statistically significant (*p*=0.528). Considering both types (gr/gr and b1/b3) of partial deletions, no statistically significant difference (*p*=0.321) could be found between the two groups.

**Figure 4.**
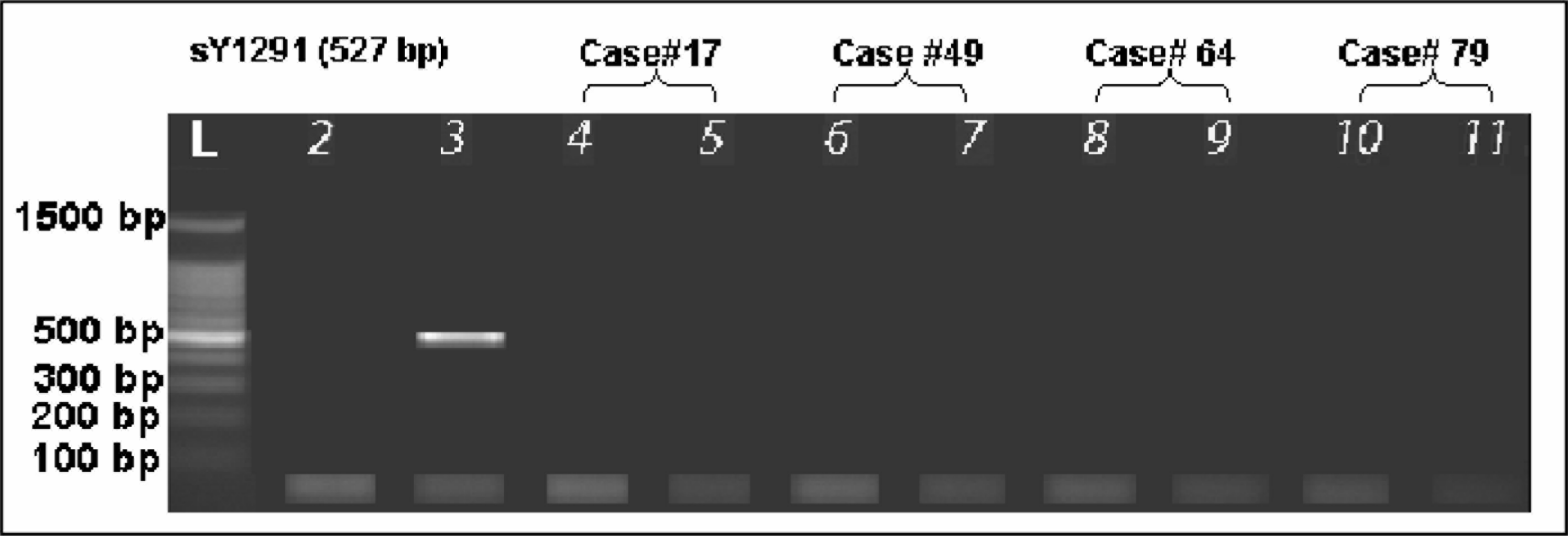
Ethidium bromide-stained agarose gel for determination of gr/gr deletion source (inherited vs. *de novo*). L: 100 bp DNA Ladder, Lane 2: negative control (female DNA), Lane 3= positive control (fertile male DNA), Lane 4: case #17.

First degree relatives of four patients with the gr/gr deletion were also determined to have this particular deletion, as shown in Figure 4.

No family history of infertility was noted for study subjects with a partial *AZFc* deletion, except one (case# 79, sperm count 0.7 M/ml) whose brother was found to have severe oligozoospermia (sperm count 0.4 M/ml). This brother was also found to have the gr/gr deletion (Figure 4, lane 11). Moreover, his maternal uncle suffered from infertility but this person was not available for testing.

Lane 5: father of case# 17, Lane 6: case# 49, Lane 7: brother of case# 49, Lane 8: case# 64, Lane 9: brother of case# 64, Lane 10: case# 79, Lane 11: brother of case# 79.

## Discussion

### Y-Chromosome Classical Microdeletions

The present study identified no classic *AZF* microdeletions in the long arm of the Y-chromosome in this population of Palestinian males. This is in general agreement with some previously published studies [40–42], although varied frequencies of Y-chromosome microdeletions (range=0.75 to 35%) have been reported by others [22,23,43–53]. The variance in microdeletion frequency noted by different investigators could be attributed to several factors influencing *AZF* microdeletion status, including genetic background and Y-chromosome haplogroups, patient selection criteria, and size of study sample.

In support of the association between genetic background and Y-chromosome microdeletions, Kihaile *et al.* (2005) studied the occurrence of Y chromosomal microdeletions in two different populations, Japanese and Africans [45]. They found a prevalence of 6.2% in the Japanese goup but no Y chromosome microdeletions in Africans. Similarly, Y haplogroups seem to be a key factor in the occurrence of microdeletions in that certain haplogroups (e.g., haplogroup E) are more vulnerable to deletions than others. Indeed, certain haplogroups may confer protection (e.g., haplogroup J) against microdeletions [22,54,55].

This bizarre behavior of the different Y-chromosome haplogroups is related to the number, presence/absence and arrangement of certain DNA elements (e.g., LIPA4 element in HERV15q2) required for homologous intrachromosomal recombination leading to deletions.

It should be noted that Nebel *et al*. (2001) reported that Palestinians differ in their Y-chromosome pool from Europeans and other Middle Eastern populations [52]. They found a high proportion of Palestinians (55.2%) residing in Israel and the Palestinian Authority (West Bank) area belong to J haplogroup (which is assumed to be protective against microdeletions). This feature might explain the inability to observe classical microdeletions in our study population.

Another important factor influencing microdeletion frequency is patient selection criteria. Significantly higher frequencies of microdeletions have been reported in the setting of histologically-confirmed Sertoli cell only syndrome (SCOS), Klinefilter syndrome, and among patients with chromosomal abnormalities, variocele and cryptorchidism, and idiopathic azoospermia accompanied by elevated serum follicle-stimulating hormone (FSH) levels [23,44,47,51,56,57]. Our patient population included only one SCOS patient who proved to have gr/gr AZFc partial deletion. Patients with chromosomal abnormalities evident by GTG banding, however, were excluded from our study population. Conversely, all idiopathic infertility cases with sperm counts <10M/ml were included for evaluation. These factors may offer additional insights as to why no Y-chromosome microdeletions were found in this study population.

### AZFc Partial Deletions

The gr/gr deletion is associated with loss of about half the *AZFc* gene content, including two of the four copies of the major *AZFc* candidate gene, known as *DAZ*. Our analysis revealed this defect in 6 (4.8%) cases. Another deletion, b1/b3, is associated with a loss of nearly 1.8 Mb of the *AZFc* region and also eliminates two *DAZ* copies. This was present in 1 (0.8%) case. In terms of these deletions, there was no statistically significant difference between the experimental and control groups. The gr/gr frequency (4.8%) observed in our study is comparable to that previously reported [11,17,30].

The pathological significance of these partial deletions is not yet clear. The gr/gr deletion, described in infertile men with varying degrees of spermatogenic failure, has been proposed by some authorities as a risk factor for spermatogenic failure or oligozoospermia [17,28–30]. In our patient population gr/gr deletion was encountered in oligozoospermic, severely oligozoospermic, and azoospermic males, indicating that this deletion cannot be linked to a particular type of spermatogenic impairment. Other investigators found no association between *AZFc* partial deletions (gr/gr or b1/b3) and male infertility [11,18,31–33,49, 58–60]. Whether such partial deletions are associated with certain male lineage haplogroup(s) remains unresolved. Indeed, the b2/b3 deletion (which was not detected in our patients) has been shown to consistently occur in Y haplogroup N [39], and the gr/gr deletion has been found in association with haplogroups D2b and Q1 [17,61,62]. Therefore, the effect of partial deletion on male infertility may vary according to the Y haplogroup of the study subjects.

Although the Y haplogroup(s) of our study patients was not specifically assessed, these data (especially regarding the gr/gr deletion) suggest that this deletion could be a heritable polymorphism rather than a *de novo* arrangement.

This is because the incidence of this deletion was not significantly different between patients and controls. Furthermore, in three cases with gr/gr deletion, the origin of the deletion appeared to be inherited and not a *de novo* rearrangement, as their fertile first-degree relatives had the same deletion. The fourth available relative was the brother of case# 79, who showed the same deletion pattern as his brother, *i.e*., another gr/gr deletion. Considering that this individual was severely oligozoospermic, it suggests that the Y-chromosome is not a major factor in this particular case, as their maternal uncle was also infertile.

The b1/b3 partial deletion was observed in only one oligozoospermic patient (sperm count 2.6 M/ml). While interesting, this isolated observation is insufficient to make any conclusions regarding its effect on spermatogenesis. This particular deletion has been observed in both control and patient groups previously however, leading some investigators to conclude that b1/b3 is probably irrelevant to spermatogenesis [60].

In conclusion, classic Y-chromosome microdeletions were not detected in this unselected population of idiopathic oligo- and azoospermic infertile patients. Results concerning gr/gr partial deletion suggest that this pattern does not represent a risk factor for male infertility and might be considered a heritable variant in this population. Further studies are needed in order to elucidate the structure of Y haplogroup(s) prevalent here, and to explore other genetic, epigenetic and/or nutritional factors that contribute to idiopathic oligo- and azoospermia in the Gaza population.

## Figures and Tables

**Table 1:** Sequences of different primer sets employed to identify Y-chromosome microdeletions among Palestinian males.

**STS**	**Primer Sequence**	**Product Size (bp)**	**Reference**
ZFY-F	ACCRCTGTACTGACTGTGATTACAC	495	[34]
ZFY-R	GCACYTCTTTGGTATCYGAGAAAGT		
SRY-F	GAATATTCCCGCTCTCCGGA	472	[34]
SRY-R	GCTGGTGCTCCATTCTTGAG		
sY746-F	TTGACTGCTTATTCTACACAAGGC	216	[35]
sY746-R	CAGGGGAAATTGGGTTTT		
sY84-F	AGAAGGGTCTGAAAGCAGGT	326	[34]
sY84-R	GCCTACTACCTGGAGGCTTC		
sY86-F	GTGACACAGACTATGCTTC	320	[34]
sY86-R	ACACACAGAGGGACAACCCT		
DBY1-F	TATTGGCAATCGTGAAAGAC	277	[36]
DBY1-R	TGCCGGTTGCCTCTACTGGT		
sY117-F	GTTGGTTCCATGCTCCATAC	261	[37]
sY117-R	CAGGGAGAGAGCCTTTTACC		
sY125-F	GGGATAGGGAAAGGGTACAA	200	[38]
sY125-R	CCGGGAGAAAAAAAACTGAA		
sY127-F	GGCTCACAAACGAAAAGAAA	274	[34]
sY127-R	CTGCAGGCAGTAATAAGGGA		
sY131-F	ACATATCCCTTGCCACTTCA	143	[38]
sY131-R	TCAGGTACCTTCTGCCTGAG		
sY134-F	GTCTGCCTCACCATAAAACG	301	[34]
sY134-R	ACCACTGCCAAAACTTTCAA		
sY152-F	AAGACAGTCTGCCATGTTTCA	125	[38]
sY152-R	ACAGGAGGGTACTTAGCAGT		
sY254-F	GGGTGTTACCAGAAGGCAAA	380	[34]
sY254-R	GAACCGTATCTACCAAAGCAGC		
sY255-F	GTTACAGGATTCGGCGTGAT	123	[34]
sY255-R	CTCGTCATGTGCAGCCAC		
sY272-F	GGTGAGTCAAATTAGTCAATGTCC	95	[14]
sY272-R	CCTTACCACAGGACAGAGGG		
sY1191-F	CCAGACGTTCTACCCTTTCG	385	[8]
sY1191-R	GAGCCGAGATCCAGTTACCA		
sY1291-F	TAAAAGGCAGAACTGCCAGG	527	[8]
sY1291-R	GGGAGAAAAGTTCTGCAACG		

**Table 2:** Partial *AZFc* deletion classification scheme employing the sY1191 and sY1291 STSs.

**AZFc deletion pattern**	**sY1291**	**sY1191**
No deletion	+	+
gr/gr deletion	−	+
b2/b3 deletion	+	−
b1/b3 deletion	−	−

(+)= No deletion; (−)= deletion

**Table 3:** Frequency of partial AZFc deletions in study and control groups.

**Group**	**n**	**gr/gr**	**b2/b3**	**b1/b3**	**Total**
Study	125	6 (4.8%)	None	1 (0.8%)	7 (5.6%)
Control	50	1 (2.0%)	None	None	1 (2.0%)
*p*	-	0.409	-	0.528	0.321
